# Retinal Nerve Fiber Layer Changes Following Cataract Surgery in Patients with and Without Preperimetric Glaucoma

**DOI:** 10.3390/jcm14207255

**Published:** 2025-10-14

**Authors:** Feliciana Menna, Laura De Luca, Mattia Calabro, Alessandro Meduri, Stefano Lupo, Enzo Maria Vingolo

**Affiliations:** 1 Department of Medical-Surgical Sciences and Biotechnologies, U.O.C. Ophthalmology, Sapienza University of Rome, Via Firenze 1, 04019 Terracina, Italy; mattia.calabro@uniroma1.it (M.C.); stefanolupo@me.com (S.L.); enzomaria.vingolo@uniroma1.it (E.M.V.); 2 Department of Biomedical and Dental Science and of Morphological and Functional Images, University of Messina, 98122 Messina, Italy; laura.deluca21@gmail.com (L.D.L.); ameduri@unime.it (A.M.)

**Keywords:** preperimetric glaucoma, retinal nerve fiber layer, optical coherence tomography, cataract surgery, glaucoma diagnosis, phacoemulsification

## Abstract

**Background:** Preperimetric glaucoma (PPG) is characterized by structural optic nerve damage without detectable functional impairment. Optical coherence tomography (OCT) is increasingly utilized to monitor glaucoma, though its reliability can be compromised by lens opacities. This study investigates retinal nerve fiber layer (RNFL) thickness changes after cataract surgery in patients with and without PPG, aiming to assess potential diagnostic inaccuracies due to cataract-induced imaging artifacts. **Methods:** Thirty eyes from 30 patients undergoing cataract surgery were analyzed, divided into two groups: Group 1 (*n* = 15) without glaucoma and Group 2 (*n* = 15) with PPG diagnosed using the Global Glaucoma Staging System. RNFL thickness was measured using Spectral-Domain OCT before and one month after phacoemulsification. Statistical analysis was performed using SPSS v23.0. **Results:** Postoperative RNFL thickness increased significantly in both groups, with a greater mean change in the PPG group (mean increase: 13 µm vs. 7 µm in controls; *p* < 0.00001). The greatest changes were observed in the inferior quadrants (*p* < 0.001). Image quality improved by approximately 34% post-surgery (*p* < 0.001). Despite higher postoperative RNFL values, none of the PPG eyes were reclassified as normal. **Conclusions:** In eyes with mild nuclear cataract, lens-related signal attenuation reduces absolute RNFL values but, in this cohort, had negligible impact on structural diagnostic classification. OCT-based structural findings in early glaucoma should therefore be interpreted with caution in the presence of cataract—recognizing that measurement bias may alter thickness values without changing PPG classification. Cataract surgery improves OCT reliability and can refine subsequent glaucoma assessment.

## 1. Introduction

Glaucoma is an ocular disease characterized by optic neuropathy with specific changes in the optic disc, with or without elevated intraocular pressure [1]. Preperimetric glaucoma (PPG) is defined as an early stage of glaucomatous disease in which only structural changes detectable by OCT are present, without visual field defects [2]. The Global Glaucoma Staging System (GGSS) developed by Dr. Brusini [3] provides a framework for classifying morphological changes of the optic disc, allowing assessment of glaucoma severity based on structural criteria and staging of preperimetric glaucoma. The development and progression of cataract, as well as cataract surgery, are common events observed during the long-term follow-up of patients with glaucoma [4].

The quality of OCT imaging is affected by opacities in the optical pathway [5]. It has been demonstrated that retinal nerve fiber layer (RNFL) thickness measurements are influenced by cataracts [6,7,8,9]. The more advanced the cataract, the lower the signal quality and the thinner the recorded RNFL thickness [5]. Since cataract and glaucoma frequently coexist in the same eye, cataract can be a confounding factor in the diagnosis and follow-up of glaucoma patients using OCT, particularly in cases of PPG.

Given that accurate evaluation of RNFL alterations is central for early glaucoma detection, the presence of cataract-related signal attenuation represents a critical diagnostic challenge in PPG, where subtle structural changes must be distinguished from measurement artifacts. Previous studies, including Kok et al. (2013) [5], demonstrated that cataracts significantly reduce OCT image quality and can lead to underestimation of RNFL thickness.

PPG remains one of the most debated and challenging areas in glaucoma diagnosis. Although structural changes of the retinal nerve fiber layer and optic nerve head can be detected with high sensitivity using OCT, the absence of corresponding visual field defects makes the diagnosis controversial [10]. Some authors have emphasized that early structural changes may precede functional alterations by years, whereas others argue that these findings may represent physiological variability or artifacts, particularly in the presence of media opacities such as cataract [11,12]. The interpretation of RNFL changes is further complicated by inter-individual variability in optic disc size, age-related thinning, and differences in imaging devices [13].

As a result, the diagnosis of PPG is often based on a combination of imaging findings, risk factor evaluation, and longitudinal follow-up rather than a single test outcome. This highlights the need for a multimodal diagnostic approach, integrating OCT parameters with perimetry, optic disc evaluation, and clinical history to minimize false-positive diagnoses [14]. Understanding these diagnostic challenges is crucial, particularly in patients with coexisting cataract, where signal attenuation can exacerbate measurement errors and lead to inappropriate initiation of glaucoma therapy.

The aim of this research is to analyze RNFL measurements after cataract surgery in patients without glaucoma and in those with a diagnosis of PPG. This analysis aims to improve clinical understanding of the interaction between structural RNFL alterations and the presence of lens opacities, thereby contributing to more accurate diagnoses and more targeted treatment for patients.

## 2. Materials and Methods

This was a prospective study conducted at the Department of Ophthalmology, Sapienza University of Rome, “A. Fiorini” Hospital of Terracina. Patient enrollment and data collection occurred from February 2023 to March 2024. Written informed consent was obtained from all participants. A total of 30 eyes from 30 patients were included and divided into two groups of 15 patients each (Table 1). Group 1 included patients with cataract without glaucoma, and Group 2 included patients with cataract and a diagnosis of preperimetric glaucoma, according to the GGSS by Dr. Brusini [3]. In Group 2, 10 patients were not receiving topical hypotensive medications, whereas 5 patients were under such treatment. Lens opacities were evaluated using the Lens Opacity Classification System III (LOCS III) [15].

Inclusion criteria were as follows: age over 60 years, indication for cataract surgery, nuclear sclerosis grade 1–2 according to LOCS III, and intraocular pressure (IOP) not exceeding 22 mmHg. A signal strength of 5/10 or higher was considered acceptable. Exclusion criteria included the following: eyes with lens abnormalities other than senile cataract, macular diseases affecting macular thickness, history of previous ocular surgery or trauma, dense lenticular opacities that interfere with the signal-to-noise ratio, and optic disc abnormalities (Table 2). Patients with visually significant posterior subcapsular opacification (PSCO) were excluded, since PSCO can cause a disproportionate reduction in OCT signal quality compared to early nuclear sclerosis. Only eyes with LOCS III grade 1–2 nuclear opacities and without relevant PSCO were included.

All patients underwent a comprehensive medical history and ophthalmological examination, including Goldmann applanation tonometry, fundoscopy, and Spectral-Domain OCT (SD-OCT Optovue, Inc., Freemont, CA, USA), after pupillary dilation with 1% tropicamide eye drops. Three OCT scans were obtained, and the one with the highest signal strength was included in the study.

Peripapillary RNFL scanning using a four-quadrant protocol was employed to measure: average total RNFL thickness, average inferior quadrant RNFL thickness, average superior quadrant RNFL thickness. Scans with poor reliability were discarded and repeat scans were performed as needed.

All patients underwent standard automated perimetry at baseline, which showed no glaucomatous defects. These normal visual field results, in combination with structural OCT alterations, supported the diagnosis of preperimetric glaucoma.

Patients in both groups underwent phacoemulsification with implantation of an SN60WF intraocular lens (IOL) under local anesthesia, performed by an experienced surgeon (with more than five years of phacoemulsification experience). The postoperative pharmacological regimen included a combination of topical antibiotic and steroid drops for both groups. Postoperative intraocular pressure (IOP) was monitored at each follow-up visit.

One month after surgery, the following were recorded: mean total RNFL thickness, mean RNFL thickness in the inferior quadrants, mean RNFL thickness in the superior quadrants.

### Statistical Analysis

All analyses were performed using SPSS v23.0. Numerical variables such as age, pre- and postoperative RNFL thickness, RNFL thickness variation, IOP, and IOP variation were expressed as mean ± standard deviation (SD). The distribution of continuous variables, including age, IOP, RNFL outcomes, and pre-post differences, was evaluated with the Shapiro-Wilk test, while homogeneity of variances was assessed with Levene’s test. In addition, age was included as a covariate in an analysis of covariance (ANCOVA) model to account for its potential confounding effect on RNFL thickness. Between-group comparisons (Controls vs. PPG) were conducted using independent-samples *t*-tests, applying Welch’s correction when variances were unequal; in cases of non-normal distribution, the Mann-Whitney U test was used instead. Within each group, pre- and postoperative values were compared using paired *t*-tests, or the Wilcoxon signed-rank test when normality was not met. To explore changes in RNFL over time and between groups, a repeated-measures ANOVA was applied, with time as the within-subject factor and group as the between-subject factor. A two-tailed significance level of α = 0.05 was adopted, and exact *p*-values were reported. Where appropriate, 95% confidence intervals and effect sizes (Cohen’s d or partial η^2^) were also provided. A *p*-value < 0.05 was considered statistically significant. Given the relatively small sample size of 30 eyes, a post-hoc power analysis was performed. Based on the observed mean RNFL change of 13 µm in the PPG group and 7 µm in the control group, with a pooled standard deviation of approximately 3.5 µm, the statistical power exceeded 0.85 (α = 0.05). This indicates that the study was adequately powered to detect clinically meaningful differences in RNFL changes between groups.

## 3. Results

The sample consisted of 30 patients recruited for the study, who presented the following demographic characteristics. The mean age in Group 1 (patients without glaucoma) was 75.3 ± 4.8 years, while in Group 2 (patients with preperimetric glaucoma) it was 68.2 ± 4.1 years. In both groups, the age range was between 60 and 80 years. Regarding gender distribution, Group 1 included 46% males (7 patients) and 54% females (8 patients), whereas Group 2 consisted of 40% males (6 patients) and 60% females (9 patients).

**Figure 1:**
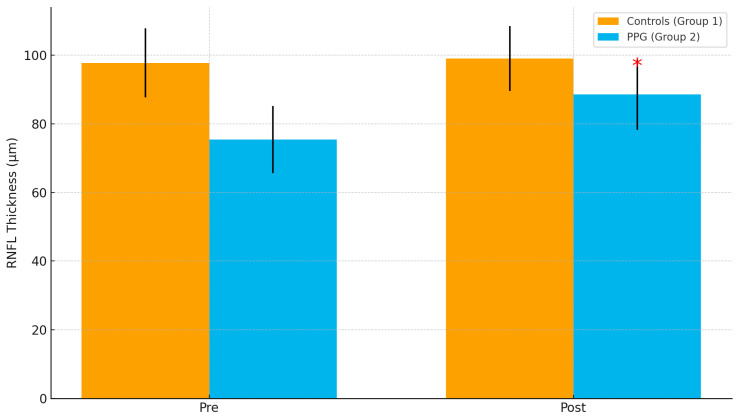
Total retinal nerve fiber layer (RNFL) thickness before and after cataract surgery in controls (Group 1) and preperimetric glaucoma patients (Group 2). Postoperative RNFL significantly increased in the PPG group, particularly in the inferior quadrants. Error bars indicate standard deviations. * *p* < 0.05.

Shapiro–Wilk tests indicated that all continuous variables were approximately normally distributed (all *p* > 0.05); parametric tests were therefore applied as prespecified. The comparison between the two groups revealed that the mean variation in RNFL thickness one month after cataract surgery was significantly greater in eyes with preperimetric glaucoma than in non-glaucomatous eyes (*p* < 0.00001). The most substantial changes in RNFL thickness were observed in the inferior quadrants, an area known to be a significant predictor of glaucoma progression (Figure 1). Additionally, the quality of the OCT images improved by 34% (*p* < 0.001). Repeated measures ANOVA confirmed a significant interaction between group (PPG vs. controls) and time (pre vs. post) (F = 14.2, *p* < 0.001). When age was introduced as a covariate, the group × time interaction remained significant (F = 13.5, *p* < 0.001), indicating that the observed RNFL changes were not attributable to age differences between groups.

In Group 1 (patients without glaucoma), the preoperative mean RNFL thickness was 97.8 ± 10.1 µm. Specifically, the mean thickness in the inferior quadrants was 104.5 ± 8.3 µm, while in the superior quadrants it was 99.3 ± 7.2 µm. One month after surgery, there was an average increase in total RNFL thickness of approximately 7 microns, reaching 104.4 ± 9.2 µm. The mean RNFL thickness in the inferior quadrants increased to 111.6 ± 6.4 µm, and in the superior quadrants to 108.1 ± 5.6 µm (Table 2 and Table 3).

In Group 2 (patients with preperimetric glaucoma, GGSS stage 1–2), the preoperative mean total RNFL thickness was 75.4 ± 9.8 µm. The mean RNFL thickness in the inferior quadrants was 83.3 ± 7.5 µm, and in the superior quadrants it was 79.5 ± 8.3 µm. Following cataract surgery, there was a significant increase in RNFL thickness, with the total mean rising by approximately 13 microns to 88.3 ± 9.3 µm. In the inferior quadrants, the RNFL thickness increased to 96.9 ± 6.1 µm, and in the superior quadrants to 92.7 ± 7.2 µm (Table 3 and Table 4).

**Figure 2:**
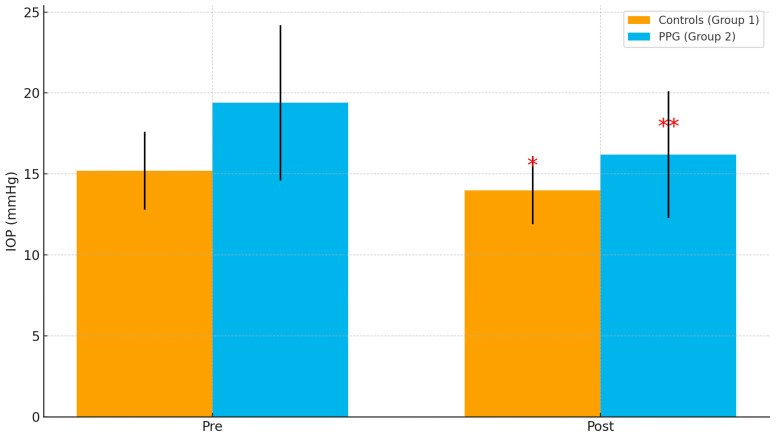
Intraocular pressure (IOP) before and after cataract surgery in controls (Group 1) and preperimetric glaucoma patients (Group 2). Significant reductions were observed in both groups, with a greater decrease in the PPG group. Error bars indicate standard deviations. * *p* < 0.05; ** *p* < 0.01.

The analyses revealed significant differences in the mean RNFL thickness in the group of patients with preperimetric glaucoma (*p* < 0.00001) compared to the group of patients without glaucoma (Figure 2).

As shown in Table 5, mean preoperative IOP was significantly higher in the PPG group compared to controls (19.4 ± 4.8 mmHg vs. 15.2 ± 2.4 mmHg, *p* = 0.003). After cataract surgery, IOP decreased in both groups, with a greater reduction in the PPG group (−3.2 ± 1.8 mmHg vs. −1.2 ± 0.9 mmHg, *p* = 0.001).

Table 6 shows the RNFL results. Preoperative RNFL thickness was significantly thinner in the PPG group than in controls (75.4 ± 9.8 µm vs. 97.8 ± 10.1 µm, *p* < 0.001). Following surgery, RNFL thickness increased in both groups, with a much greater gain in PPG patients (+13.2 ± 3.5 µm vs. +6.6 ± 1.0 µm, *p* < 0.001). These findings indicate that cataract removal improves OCT image quality and RNFL measurement reliability, especially in PPG patients.

Although RNFL thickness increased significantly after surgery, none of the patients initially diagnosed with preperimetric glaucoma (PPG) were reclassified as normal postoperatively.

## 4. Discussion

The analysis of RNFL thickness following cataract surgery in patients with preperimetric glaucoma revealed significant findings that underscore the importance of thorough clinical evaluation. A glaucoma diagnosis based solely on RNFL alterations, especially when these changes are mild, has been a subject of scientific debate [16,17]. The results of this study suggest that RNFL damage observed in patients with mild structural changes associated with lens opacities can lead to a misdiagnosis of glaucoma. Approximately 70% of patients with such mild alterations showed an improvement in RNFL thickness after surgery, indicating that OCT-based interpretations in these cases may not accurately reflect the true condition of the optic nerve. Therefore, a diagnosis of glaucoma based exclusively on structural measurements is not reliable in the presence of concomitant ocular pathologies. Because cataract-related signal attenuation biases OCT downward, postoperative RNFL increases may reflect correction of measurement error rather than true neuroaxonal thickening. A key clinical question is whether such bias alters diagnostic classification; in our cohort, classification remained stable despite higher postoperative RNFL values indicating that for LOCS III 1–2 nuclear cataracts, measurement bias reduces absolute RNFL values but has negligible impact on PPG classification. Furthermore, after adjusting for age, the differences in RNFL variation between groups persisted, confirming that age did not significantly bias the results.

Our findings are in agreement with Kok et al. (2013) [5], who reported that cataract optical density reduces OCT signal quality and leads to underestimation of RNFL thickness. Similarly, Savini et al. (2006) [18] highlighted that cataract influences Stratus OCT measurements, while Rao et al. (2011) [19] showed that spectral-domain OCT values are affected in the presence of cataract. In line with these reports, we observed a significant increase in RNFL values after cataract extraction, particularly in the PPG group, reflecting an improvement in imaging quality rather than a true structural thickening. Jo et al. (2016) [20] also reported longitudinal increases in RNFL thickness following cataract surgery in glaucoma patients, supporting our results. Lee et al. (2012) [21] and Jha et al. (2017) [22] described similar postoperative RNFL improvements, reinforcing the evidence that cataract removal enhances OCT reliability. This comparison strengthens the clinical significance of our results. These findings indicate that, despite the improvement in OCT image quality and the significant increase in RNFL thickness after cataract removal, the postoperative changes were not sufficient to alter the PPG diagnosis. This suggests that mild cataracts can artifactually reduce RNFL values but rarely result in misclassification of normal eyes as glaucomatous.

Cataract surgery has been shown to improve OCT image quality and RNFL measurements, particularly in eyes with pre-existing media opacities [23]. Several studies demonstrated that cataract extraction not only enhances the reliability of structural imaging but can also increase apparent RNFL thickness values, thereby reducing the risk of overestimating glaucomatous damage [18,20,21]. This emphasizes the confounding role of lens opacity, especially in early disease stages where diagnostic uncertainty is greater. These findings are consistent with the known optical properties of cataracts, which scatter and absorb light, reducing OCT signal strength and leading to underestimation of RNFL thickness. After phacoemulsification, improved optical clarity enhances image quality, resulting in more reliable segmentation and thicker RNFL measurements. Importantly, this apparent increase may represent a correction of measurement error rather than a true biological thickening of the RNFL. Moreover, the interaction between cataract and glaucoma should not be underestimated. Cataract-related changes may mask or mimic glaucomatous damage, complicating disease staging and follow-up [19,22]. For this reason, the interpretation of OCT findings should always be contextualized with functional assessments such as perimetry, alongside clinical evaluation of the optic disc and intraocular pressure.

One limitation of this study concerns patient selection. The exclusion of patients with advanced lens opacities may have introduced selection bias, as patients at different stages of glaucoma may respond differently to cataract surgery. This limitation restricts the generalizability of the findings, and future studies should include a broader range of cataract and glaucoma stages. Another limitation is the short follow-up period. Assessing RNFL thickness only one month postoperatively may not capture long-term changes; thus, extended follow-up is necessary to better understand temporal dynamics [24,25]. Additionally, while some clinical variables were controlled, other potential confounding factors—such as patient age, family history of glaucoma, and concurrent pharmacologic treatments—were not accounted for, which may influence the results. The age discrepancy between groups (63.4 ± 8.2 years in controls vs. 66.7 ± 7.9 years in PPG patients) represents a potential confounding factor, since RNFL thickness declines naturally with age at a rate of approximately 0.2–0.4 µm per year [17]. This difference could partially account for the thinner baseline RNFL observed in the PPG group and must be considered when interpreting group comparisons. Other potential confounding factors such as systemic hypertension, diabetes mellitus, family history of glaucoma, and central corneal thickness were not collected and represent limitations of this study.

This study highlights the need for a holistic diagnostic approach in ophthalmology. Accurate diagnosis of PPG requires a combination of structural assessments, clinical evaluations, and patient history [26,27,28]. Reliance on a single parameter, such as RNFL thickness, may result in misclassification and inappropriate management [29,30]. Further investigation into the complex interactions between lens opacity and glaucomatous changes is essential to improve clinical management and patient quality of life. Prospective studies with larger populations and long-term follow-up will be critical to validate these findings and to refine diagnostic strategies for patients with coexisting cataract and glaucoma.

## 5. Conclusions

In conclusion, cataract surgery should not be regarded solely as a vision-restoring procedure, but also as a strategic opportunity to refine glaucoma diagnosis and tailor long-term management, especially in patients with coexisting lens opacity and suspected PPG. In eyes with LOCS III grade 1–2 nuclear cataract, phacoemulsification improves OCT signal quality and increases measured RNFL thickness. However, in our cohort, no PPG eye was reclassified as normal postoperatively, indicating that cataract-related attenuation affects absolute RNFL values more than structural diagnostic classification. Clinicians should interpret OCT measurements in early glaucoma with awareness of this measurement bias and confirm structural findings with multimodal assessment.

## Figures and Tables

**Table 1:** Demographic and baseline characteristics of study groups.

Variable	Controls (*n* = 15)	PPG ^1^ (*n* = 15)	*p*-Value
Age (years, mean ± SD)	63.4 ± 8.2	66.7 ± 7.9	0.312
Gender (M/F)	7/8	8/7	0.715
Pre-op IOP ^2^ (mmHg, mean ± SD)	15.2 ± 2.4	19.4 ± 4.8	0.003
Pre-op RNFL ^3^ (µm, mean ± SD)	97.8 ± 10.1	75.4 ± 9.8	<0.001

^1^ PPG= Preperimetric glaucoma; ^2^ IOP = Intraocular pressure; ^3^ RNFL = Retinal nerve fiber layer.

**Table 2:** Inclusion and exclusion criteria.

Inclusion Criteria	Exclusion Criteria
BCVA ^1^ not lower than 20/40	History of previous ocular surgery or correction of moderate-to-high ametropia
Cataract stage 1 or 2 according to LOCS ^2^ III	Presence of concomitant ocular diseases or systemic conditions affecting vision
Absence of glaucomatous visual field defects	Advanced lens opacities (stage 3 or 4 according to LOCS III)
PSD ^3^ index ≤ 2.5 and MD ^4^ ≥ −4 (Global Glaucoma Staging System)	
Refractive errors within ±5 diopters	
IOP ^5^ ≤ 22 mmHg	

^1^ BCVA = Best-corrected visual acuity; ^2^ LOCS = Lens Opacity Classification System; ^3^ PSD = Pattern standard deviation; ^4^ MD = Mean deviation; ^5^ IOP = Intraocular pressure.

**Table 3:** Baseline characteristics before surgery.

Parameter	Group 1 (Controls, *n* = 15)	Group 2 (PPG ^1^, *n* = 15)	*p*-Value
Age (years)	63.4 ± 8.2	66.7 ± 7.9	0.312 ^a^
Gender (M/F)	7/8	6/9	0.72 ^b^
Pre-op IOP ^2^ (mmHg)	15.2 ± 2.4	19.4 ± 4.8	0.003 ^a^
Mean total RNFL ^3^ (µm)	97.8 ± 10.1	75.4 ± 9.8	<0.001 ^a^
Superior quadrant RNFL (µm)	99.3 ± 7.2	79.5 ± 8.3	<0.001 ^a^

^1^ PPG = Preperimetric glaucoma; ^2^ IOP = Intraocular pressure; ^3^ RNFL = Retinal nerve fiber layer. Values are presented as mean ± standard deviation (SD) unless otherwise indicated. ^a^ Independent-samples *t*-test (Welch’s correction if variances unequal). ^b^ Fisher’s exact test.

**Table 4:** Findings one month after surgery.

Parameter	Group 1 (Controls, *n* = 15)	Group 2 (PPG ^1^, *n* = 15)	*p*-Value
Post-op IOP ^2^ (mmHg)	14.0 ± 2.1	16.2 ± 3.9	0.041 ^a^
Mean total RNFL ^3^ (µm)	104.4 ± 9.2	88.3 ± 9.3	0.002 ^a^
Superior quadrant RNFL (µm)	108.1 ± 5.6	92.7 ± 7.2	<0.001 ^a^
Inferior quadrant RNFL (µm)	111.6 ± 6.4	96.9 ± 6.1	<0.001 ^a^

^1^ PPG = Preperimetric glaucoma; ^2^ IOP = Intraocular pressure; ^3^ RNFL = Retinal nerve fiber layer. Values are presented as mean ± standard deviation (SD). ^a^ Independent-samples *t*-test (Welch’s correction if variances unequal).

**Table 5:** Changes in intraocular pressure (IOP) before and after cataract surgery.

Parameter	Controls (*n* = 15)	PPG ^1^ (*n* = 15)	*p*-Value
Pre-op IOP ^2^ (mmHg, mean ± SD)	15.2 ± 2.4	19.4 ± 4.8	0.003
Post-op IOP (mmHg, mean ± SD)	14.0 ± 2.1	16.2 ± 3.9	0.041
Δ IOP (mmHg, mean ± SD)	−1.2 ± 0.9	−3.2 ± 1.8	0.001

^1^ PPG = Preperimetric glaucoma; ^2^ IOP = Intraocular pressure.

**Table 6:** Retinal nerve fiber layer (RNFL) thickness before and after cataract surgery.

Parameter	Controls (*n* = 15)	PPG ^1^ (*n* = 15)	*p*-Value
Pre-op RNFL ^2^ (µm, mean ± SD)	97.8 ± 10.1	75.4 ± 9.8	<0.001
Post-op RNFL (µm, mean ± SD)	104.4 ± 9.5	88.6 ± 10.3	0.002
Δ RNFL (µm, mean ± SD)	+6.6 ± 1.0	+13.2 ± 3.5	<0.001

^1^ PPG = Preperimetric glaucoma; ^2^ RNFL = Retinal nerve fiber layer.

## Data Availability

The original contributions presented in this study are included in the article. Further inquiries can be directed to the corresponding author.

## Abbreviations

The following abbreviations are used in this manuscript:

PPG	Preperimetric glaucoma
RNFL	Retinal nerve fiber layer
OCT	Optical coherence tomography
GGSS	Global Glaucoma Staging System
LOCS	Lens Opacity Classification System
IOP	Intraocular pressure
IOL	Intraocular lens
